# Improvement of the signal to noise ratio for fluorescent imaging in microfluidic chips

**DOI:** 10.1038/s41598-022-23426-z

**Published:** 2022-11-07

**Authors:** Xiaocheng Liu, Hanliang Zhu, Ján Sabó, Zdeněk Lánský, Pavel Neužil

**Affiliations:** 1 Department of Microsystems Engineering, School of Mechanical Engineering, Northwestern Polytechnical University, 127 West Youyi Road, Xi’an, 710072 Shaanxi People’s Republic of China; 2 Institute of Biotechnology, Czech Academy of Science, Průmyslová 595, 252 50 Vestec, Czech Republic; 3 Department of Physical Chemistry, Faculty of Science, Charles University, Hlavova 8, 12800 Prague 2, Czech Republic

**Keywords:** Engineering, Biomedical engineering, Imaging, Fluorescence imaging

## Abstract

Microfluidics systems can be fabricated in various ways using original silicon glass systems, with easy Si processing and surface modifications for subsequent applications such as cell seeding and their study. Fluorescent imaging of cells became a standard technique for the investigation of cell behavior. Unfortunately, high sensitivity fluorescent imaging, e.g., using total internal reflection fluorescence (TIRF) microscopy, is problematic in these microfluidic systems because the uneven surfaces of the silicon channels’ bottoms affect light penetration through the optical filters. In this work, we study the nature of the phenomenon, finding that the problem can be rectified by using a silicon-on-insulator (SOI) substrate, defining the channel depth by the thickness of the top Si layer, and halting the etching at the buried SiO_2_ layer. Then the fluorescent background signal drops by = 5 times, corresponding to the limit of detection drop from = 0.05 mM to = 50 nM of fluorescein. We demonstrate the importance of a flat surface using TIRF-based single-molecule detection, improving the signal to a noise ratio more than 18 times compared to a conventional Si wafer. Overall, using very high-quality SOI substrates pays off, as it improves the fluorescence image quality due to the increase in signal-to-noise ratio. Concerning the cost of microfluidic device fabrication—design, mask fabrication, wafer processing, and device testing—the initial SOI wafer cost is marginal, and using it improves the system performance.

## Introduction

With the advent of micromachining, microfluidics technology also became a common technique, starting with chemical and biochemical reactors, sample sorting, mixing, and biosensing. There are several techniques for sensing: electrochemical methods such as voltammetry, galvanometry^1^, or impedance spectroscopy^2^, and electrical methods such as impedance measurement^3^ and mass detection using external mass spectroscopy^4^ or internally either surface acoustic waves^5^ or quartz crystal microbalance^6^. They all require the implementation of electrodes inside the microfluidic channels. Of course, it is possible, but it makes the device fabrication process more complicated—a problem for disposable chips.

In principle, optical methods have advantages compared to electrical. The optics will never contact the analyte, so there is no need for disposal to eliminate sample-to-sample cross-contamination. Colorimetry^7^ and turbidity^8^ measurements are simple methods but their limit of detection (LOD) rather high preventing them to be commonly used. The methods based on plasmon generations, such as surface plasmon resonance (known as SPR)^9^ or localized surface plasmon resonance (known as LSPR)^10^, are more complex. The first requires a gold-coated substrate placed on a rotating prism with a laser detecting the minimal incident angle; the second uses an easier setup but requires determining the spectrum by use of a spectrum analyzer^11^ reflected from the nanostructured surface. Unfortunately, these methods all have one feature in common, which is relatively high noise that leads to the insufficient (LOD) for numerous applications.

Nevertheless, there are a few optical techniques commonly used with a sufficiently low level of LOD such as electrochemiluminescence (ECL)^12^ and fluorescence^13^. The ECL method requires an electric field-triggered redox reaction, either with an external power supply concerning the electrodes^14^ or by using free-floating metal platelets large enough for the applied external electric field to reach at least 1 V voltage drop in the vicinity of the platelets^15^. These methods have one great advantage of having almost no light in the background, as there is no external light required. Fluorescence-utilizing methods are based on the excitement by illumination, using lower wavelengths to excite electrons from an orbit and detecting relaxation to a lower energy state, exhibiting light generated with a longer wavelength than the one that was used for excitation. The exciting light historically originated using either Ar laser or Hg and Xe lamps. All those light sources are multispectral and the user has to select correct wavelengths, such as 488 nm from the Ar laser source or one of ultraviolet (UV) i-line (365 nm), h-line (405 nm), or g-line (436 nm), or longer wavelengths of 488 nm, 546 nm or 579 nm from Hg and Xe lamps, using appropriate optical filters to block the other wavelengths.

Typical materials are fluorescein^16^ and rhodamine 6G^17^, which excite with the highest efficiency excitement wavelength of = 490 nm (pH-dependent) and 570 nm (solvent-dependent), and the highest power of emission wavelength of 515 nm and from 580 to 590 nm, respectively. Apart from the basic fluorophores, there are a large number of others for various purposes such as chemical and biochemical analysis monitoring, DNA detection using real-time polymerase chain reaction (qPCR)^18^, digital polymerase chain reaction (dPCR)^19^, enzyme-linked immunosorbent assay (ELISA)^20^ and cell imaging^21^. Here, fluorescence plays an indispensable role; researchers can greatly enhance the cell contrast as well as the parts, using a live-dead assay to identify ratios between live and dead cells^22^.

There is a phenomenon preventing high-resolution microscopy inside microfluidic devices such as autofluorescence of plastic materials used for microfluidic chip fabrication. We noticed a similar problem in our chips made of silicon capped with glass with an anodic bonding; clearly, no materials were exhibiting significant autofluorescence, if any. The high-end filters are based on interference by multilayer thin films; thus, they are sensitive to light incidence angles as it is shown in Semrock’s website in using *MyLightTool* application available there^23^. In this work, we performed a detailed study of microfluidics to discuss the origin of the background noise and the method of suppressing it by two orders of magnitude allowing more sensitive fluorescence measurement as well as he total internal reflection microscopy to be used with its maximum resolution (TIRF)^22^.

## Optical filter measurement and discussion

### Fluorescence filters

The typical fluorescent optical system consists of: a light source; an excitation filter (XF) to keep unwanted components from exciting light and possibly interfering with the results; a dichroic mirror (DM) to reflect filtered excitation light on the specimen; and an emission filter (MF) to block filtered exciting light, allowing only the emitted light to pass to the optical detector, which can be a photodiode, photomultiplier tube (PMT), or a suitable camera.

**Figure 1:**
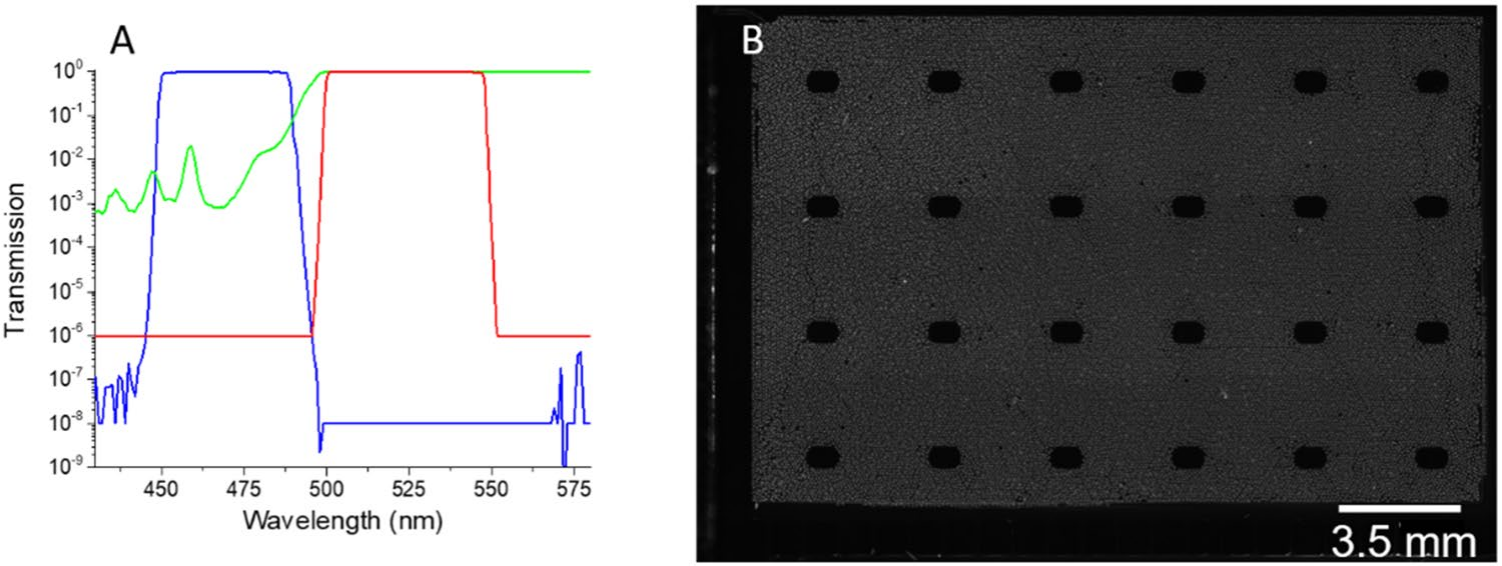
(**A**) Fluorescein isothiocyanate (FITC) filter set transmission showing properties of the excitation filter (blue), dichroic mirror (red), and emission filter (green). Both excitation and emission filters have a light incident angle of 0° while the dichroic mirror is 45°. (**B**) A fluorescent image of a digital polymerase chain reaction (dPCR) chip from Stilla^24^ using an FITC filter set. The droplets contained precipitated salts from the buffer exhibiting false fluorescent signal.

Here we used high-end optical filters, ET series model 49,002 (Chroma Technology Corp., Bellows Falls, VT, USA) intended for the FITC type of fluorescence^25^. The filters have excellent parameters, with the nearly 100% of transmission in the desired band from 450 to 488 nm and from 500 to 548 nm, with an excellent raise and falling edge of 1.478 and − 1.563 nm dec^−1^ and 1.524 and − 0.899 nm dec^−1^ for XM and MF, respectively (Fig. 1A). Their excellent properties are achieved by the interference effect, using multiplayer coatings having up to 150 layers of ET series. Unfortunately, that is only acceptable for light perpendicular to the filter surface, i.e., for an incident angle close to 0°. Once this angle significantly deviates from 0°, the properties of the filters change due to the different lengths of the light optical path^26^. Typically, that is not a big problem for transmission light-based fluorescence measurement. However, reflection light-based observation and imaging is sensitive to light scattering due to dispersion at surface topography, such as in the precipitated crystals of buffer solution in the digital polymerase chain reaction chip (Fig. 1B). Also surface roughness affecting the fluorescent imaging is known from previous fluorescent spectroscopy measurement^27^ including total reflection fluorescence^28^.

Recently, the precise model of a thin-film interference filter has been conducted showing its property’s sensitivity to the angle of incident light^29^. It is well known effect which can be simulated by a specialized optical software as well as using online tools such as Semrock’s MyLightTool^23^. Unfortunately, Chroma Optical does not offer these details of the sputtered layers in public domain thus we had to measure it.

### Filter angle testing setup and results

**Figure 2:**
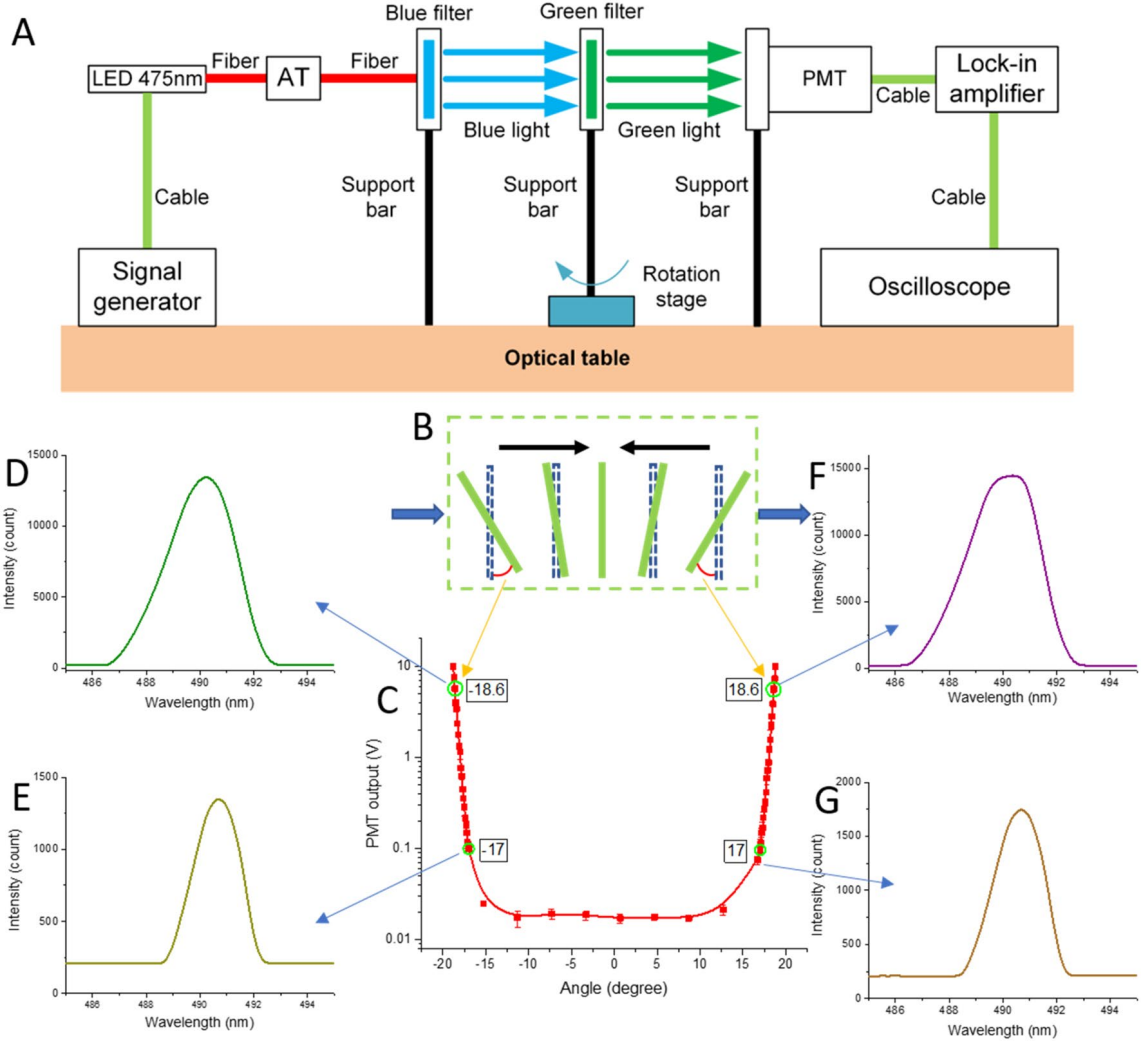
(**A**) Diagram of the light measurement through the rotated filter showing LED with a principal wavelength of 475 nm (LED 475 nm) connected to the excitation filter (blue) perpendicular to the light via optical fiber and attenuator (AT), emission filter (green) on a rotating platform, and either a photomultiplier tube (PMT) as light detector (shown here) or spectrum analyzer (not shown here). (**B**) Schematically showing the green filter rotation and its influence on (**C**) measured detected power on the PMT and (**D**–**F**) detected spectra for four different angles of the emission filter position.

We used a light-emitting diode (LED) with a principal wavelength of 475 nm as a light source. This light was filtered by XM to block light with a wavelength longer than 490 nm, and after passing through FM mounted on a rotating platform, the transmitted light was detected either by a spectral analyzer or by a PMT through an optical fiber system. In principle, there should be no significant power of light passing through the system when the incident light angle at both filters is 0°. We measured this light spectrum penetrating through the system as a function of the incident angle at the FM filter (Fig. 2). The optical spectrum was measured directly using the LED DC power with 20 mA of electrical current. We shielded the system from the ambient to avoid unwanted interference.

The PMT setup was different. We powered the LED using = 10 mA of electrical current and set its modulated power at a frequency of 1.012 kHz using a signal generator. The PMT was set with minimal gain, having 0.5 V voltage at the gain control input. The PMT output signal was demodulated by a lock-in amplifier with sensitivity set to 1 V scale^−1^, with a time constant set to 100 ms. As we used a lock-in amplification system, optical path shielding from ambient light was not essential.

Initially, the light source, filters, and detector were adjusted to be collinear. We then rotated the green filter to reach the maximal amplitude value of the PMT output and recorded the response as well as the angle value of = 9.78 V and = 318.5°, respectively (Fig. 2A). The amplitude value was decreased, and the angle was decreased in steps of 0.1° and recorded until it plateaued. The same measurement was done on the other rotation side, starting with a similar amplitude of = 9.84 V according to the angle of = 356.2°. We then shifted the center of the curve to zero by calculating the offset of the measurement using a linear curve fitting in a descending stage (Fig. 2B). The spectrum of the changing rotation angles was also measured (Fig. 2C–G). We found that the filter maintains its properties in a range of incident angles from = − 15 to = 15°. Once the absolute value of an incident angle is greater than = 15°, light that is normally blocked begins to penetrate through the filter, resulting in overall power increases of transmitted light.

The thin-film interference filter is sensitivity to the angle of incident light resulting in the rough surface more fluorescence noise. We provide the solution by chip fabrication based on SOI wafer and compared the fluorescence signal from the same structure on different substrates.

## Methods

### Chip design and fabrication

**Figure 3:**
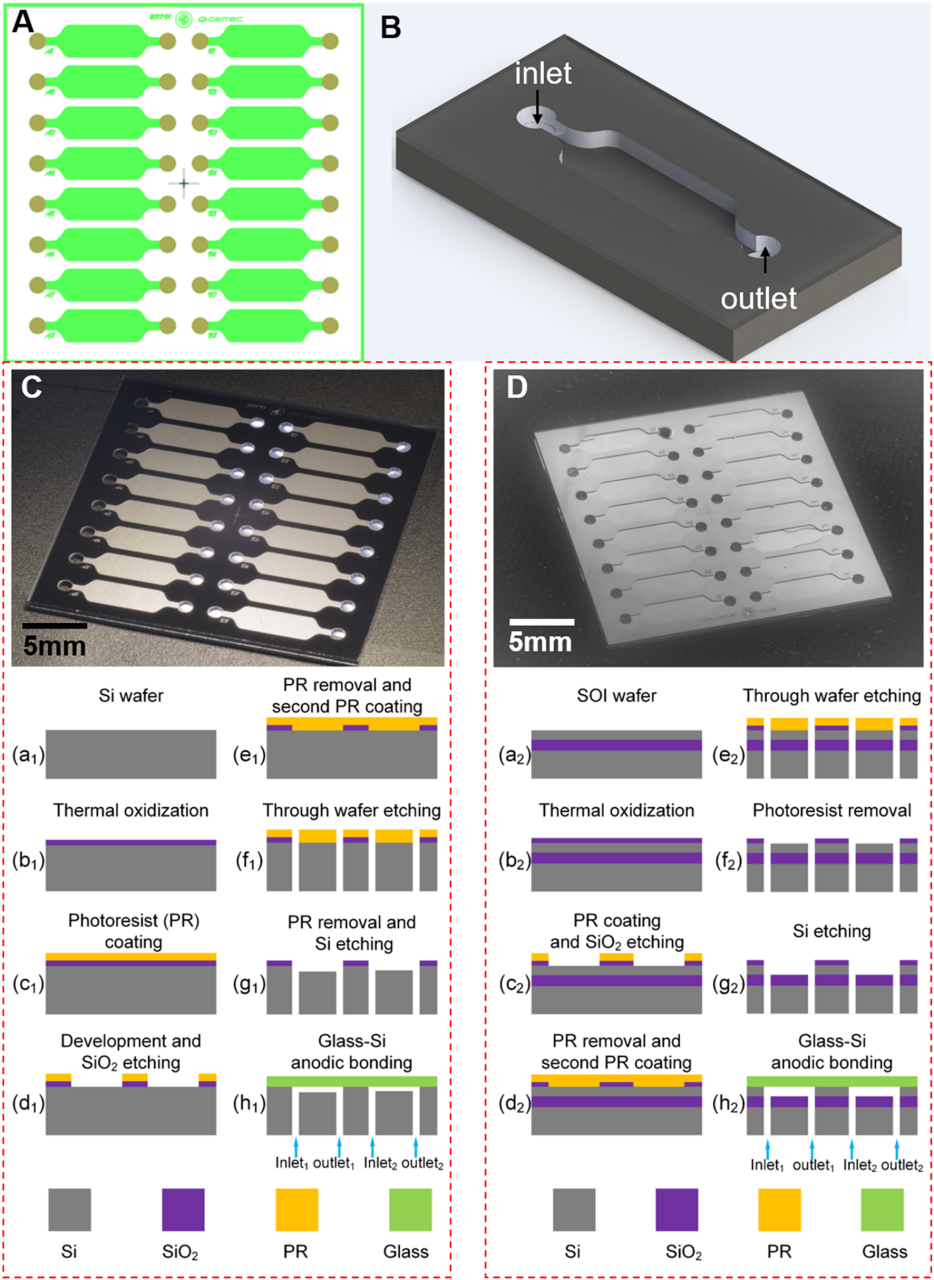
Description of the microfabrication processes based on different surface. (**A**) Layout of the chip with individual chamber for fluorescence measurement and (**B**) its visualization using computer-aided software. (**C**) Chip fabricated using conventional Si and (**D**) SOI substrate.

We designed our chips (Fig. 3A) using Nanolithography Toolbox^30^, having 16 chambers with tangential tube connections^31^, each with an area of = 15 mm^2^, a depth of = 100 µm, and a volume of = 1.5 nL, and visualized a single chamber using computer-aided software (Fig. 3B). We used our conventional microfabrication technique for microfluidic chips, consisting of patterned Si substrate using two lithography steps and a non-patterned glass cover anodically bonded to the Si substrate^32^. We conducted both lithography steps on a *flat* substrate giving us no additional challenges, such as performing lithography on a substrate with significant topography changes. Then we used the Bosch process with deep reactive ion etching (DRIE) based on a combination of SF_6_ etching and C_4_F_8_ polymer deposition. This is a well-known process with an excellent etch rate of a few µm min^−1^^33^. The etched Si wafer was then capped using anodic bonding, an easy task since there was no pattern on the glass substrate, meaning no strict alignment rules had to be followed.

The fabrication was first conducted using conventional Si wafers with a diameter of = 100 mm (Fig. 3C-a1). The wafers were oxidized in a furnace using a wet O_2_ environment to grow a SiO_2_ layer with a target thickness of 100 nm (Fig. 3C-b1). Then the wafers were spin-coated with a photoresist (Fig. 3C-c1), and the first lithography was performed defining the chambers and the channels. After the photoresist development, the SiO_2_ layer was etched using a reactive ion etching of a CHF_3_/O_2_ gas mixture (Fig. 3C-d1). The photoresist was removed, the wafers were cleaned, and we spin-coated a second photoresist layer—this time with a thickness between 10 and 12 µm (Fig. 3C-e1). After exposure and development, the wafers were etched by DRIE through the entire Si substrate to form the access holes for fluid inlets and outlets (Fig. 3C-f1). Then the photoresist was removed, and a second DRIE step was conducted for the 100 µm depth required for the chamber. This time, the original patterned SiO_2_ served as a mask (Fig. 3C-g1). The masking SiO_2_ layer was removed by wet etching using HF/NH_4_F with concentrations of 49% and 40%, and the ratio between them of 1:6. The last fabrication step was thermal oxidation of the wafers at a set temperature of 900 °C to grow a 5 nm layer of thermal SiO_2_ to define the chamber surface properties. Finally, the Si wafers were anodically bonded (Fig. 3C-h1) with Corning 7440 glass substrate with a diameter of = 100 mm and a thickness of = 170 µm (Fig. 3C), as the TIRF microscope objective lens is optimized for this glass thickness.

Once the wafers were diced into individual chips (Fig. 3C), they were coated with FAS-17 fluorosilane using a combination of wet incubation and chemical vapor deposition at a lower pressure at an elevated temperature, leading to self-assembly monolayers with excellent properties such as contact angles higher than 105°^32,34^. We also performed identical fabrication using an SOI substrate diameter of 100 mm, a buried SiO_2_ thickness of 100 nm, and a top Si layer of 100 µm. The process is schematically represented at (Fig. 3Ca2–h2). The only difference between fabrication using conventional and SiO_2_ substrates was the upfront definition of the chamber depth of the SOI substrate, dictated by the top Si layer thickness, while for the conventional substrate the depth was determined by the etching time of the second DRIE. Also, the DRIE of the access holes of the SOI wafer is more complex, as the etching using the conventional Bosch process had to be interrupted once the buried SiO_2_ layer was reached to change into SiO_2_ etching; once through, we continued with the Bosch process to finish etching the Si (Fig. 3D).

### TIRF setup

#### Microtubule preparation

Taxol stabilized microtubules were prepared as described previously^35^. We used 5 μL of a porcine brain tubulin mixture, having 4 mg mL^−1^ with 80% unlabeled and 20% fluorescently labeled by Atto647 (Cytoskeleton, Inc.; Denver, CO, USA), incubated with 1.25 μL of polymerization mix having 25% DMSO in 20 mM MgCl_2_ and 5 mM GTP in BRB80, at 37 °C for 30 min. Subsequently, = 100 μL of BRB80 supplemented with 10 μM Taxol (BRB80T) was added to the polymerized microtubules before centrifugation for 30 min at 18,000 g. Pellets containing microtubules were resuspended in 100 μL of BRB80T and stored at room temperature.

#### Single-molecule motility assay

Silanized flow cells were incubated for 5 min with 20 μg·mL^-1^ anti-tubulin antibody (Sigma Aldrich, T7816, St. Louis, Missouri, USA) and subsequently blocked by 1% pluronic F127 in phosphate-buffered saline (commonly known as PBS for 1 h. Flow cells were then washed with BRB80T and incubated with microtubules for 2 min. Unbound microtubules were removed by BRB80T that were subsequently exchanged for a motility buffer. This buffer (BRB80) contained Taxol with a concentration of 10 µM, dithiothreitol with a concentration of 10 mM, D-glucose with 20 mM, 0.1% Tween-20, 0.5 mg mL^−1^ casein, 1 mM Mg-ATP, 0.22 mg mL^−1^ glucose oxidase, and 20 µg mL^−1^ catalase. For single-molecule stepping, we added to the flow cell 2.5 nM of constitutively active kinesin-1-eGFP into a motility buffer.

#### Imaging and analysis

The total internal reflection (TIRF) imaging was conducted using an inverted Nikon Eclipse Ti-E wide-field microscope equipped with a 100 × HP APO TIRF objective lens, H-TIRF module, LU-NV Laser Unit (all, Nikon, Tokyo, Japan) and a set of images creating a movie was captured by an sCMOS camera, model ORCA 4.0 V2 (Hamamatsu Photonics, Hamamatsu City, Japan). Movies were acquired for 30 s with 200 ms exposure time using NIS-Elements Advanced Research software v5.21 (Laboratory Imaging, Prague, Czech Republic). In the first frame of the series of captured images forming the movie, both HiLyte647-labelled microtubules and Kinesin-1-eGFP were imaged by sequential laser excitation via quad-band set filter model TRF89901v2 (Chroma Technology Corp., Bellows Falls, VT, USA). The remaining frames of the movie were acquired only for Kinesin-1-eGFP fluorescence to increase the frame rate of the captured movies. Signal to noise ratio of the acquired images was quantified using ImageJ software (NIST, Gaithersburg, MA, USA)^36^.

## Result and discussion

### Basic chip testing

**Figure 4:**
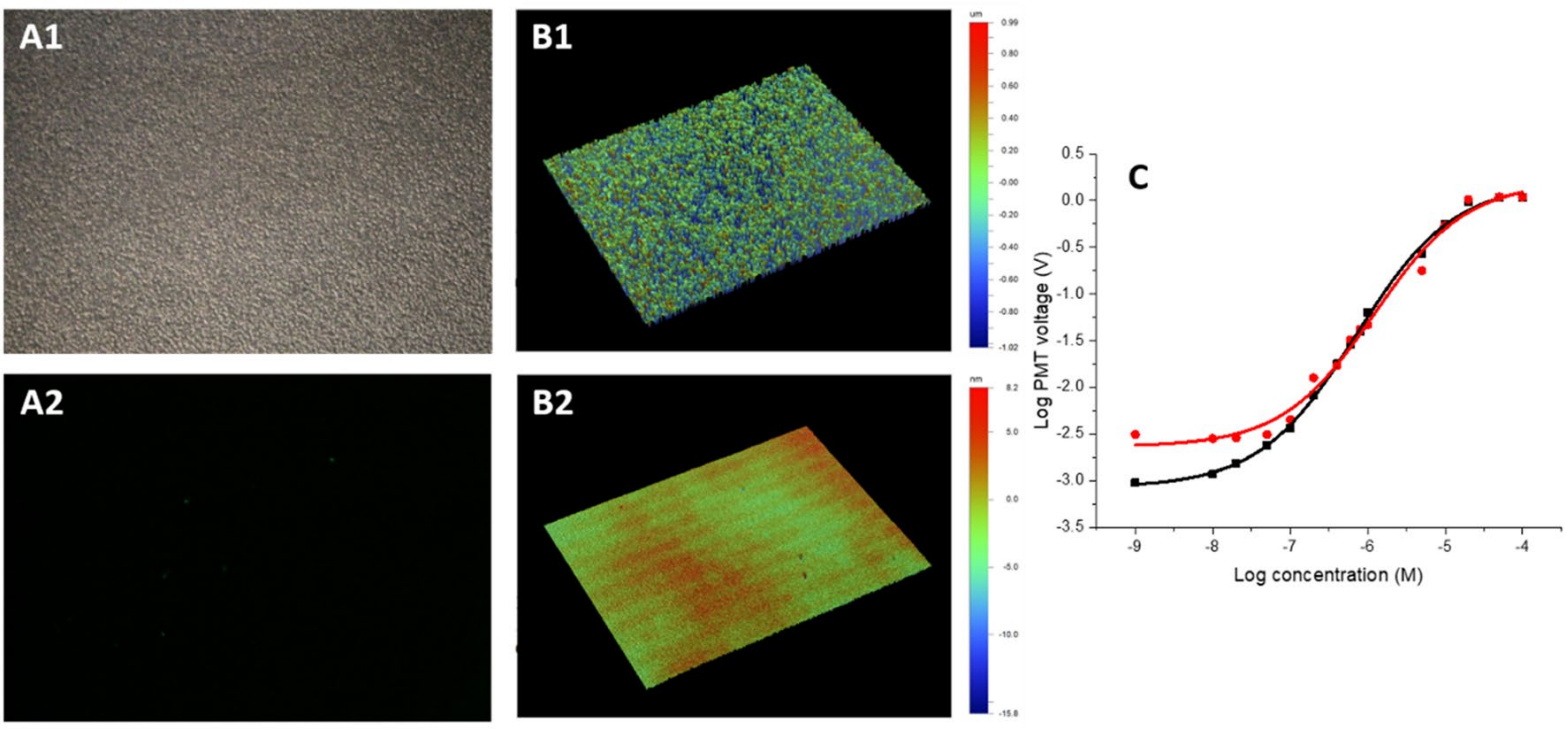
(**A**) Image of the conventional Si surface after etching using dark field microscopy with a 20 × objective lens. (**B**) Interference image of SOI (top) and standard Si (bottom) wafer chips using VSI mode. The images were processed using the auto-scale mode, with the SOI having a total scale of = 24 nm, while the conventional Si chip has a total scale = of 2.01 µm—thus = 84 × greater. (**C**) PMT voltage as a function of fluorescein concentration with the type of chamber material as a parameter using logarithmic scales for both axes; red is conventional Si substrate and black is SOI-based substrate.

We placed both chips under an optical microscope and captured dark field images of both Si chip, the one made of conventional Si (Fig. 4A1) and the second one made of an SOI substrate (Fig. 4A2). We did observe strong light scattering signal from the bottom of the chamber made a conventional Si substrate, while there was practically no scattering observed from the SOI-based chamber; it is this scattering that causes problems with the fluorescent background. We measured the surface topography using a white light optical profiler based on a Mireau interferometer^37^ equipped with a 50 × objective lens, using vertical shift interferometry mode (VSI) (Fig. 4B1,B2). The roughness of the bulk Si chip surface was 84 × greater than the SOI’s. We also used a stylus type profiler, determining the surface roughness as (76.883 ± 2.068) and (1.863 ± 0.015) nm RMS, both (mean ± standard deviation) for the standard and SOI-based chips, respectively.

### Chip testing with fluorescein

We then performed fluorescence measurement using an optical microscope equipped with an FITC filter having modulated LED with 475 nm of principal wavelength as illumination, using 10 mA of electrical current modulated at a frequency set to 1.028 kHz. The excited signal was captured by a PMT, with its gain set by the external power supply to 0.5 V, the PMT output processed by a lock-in amplifier with its sensitivity set to 1 V scale^−1^, time constant to 100 ms, and amplitude recorded by an oscilloscope.

We filled the chambers in the chips with fluorescein with solution, with the dilution starting from = 1 nM up to = 100 µM, while keeping one chamber empty for reference to determine the fluorescence LOD of each chip. Then we measured the fluorescence amplitude from all chambers and plotted it as a function of fluorescein concentration (Fig. 4C). Based on the measurement, the fluorescence LOD of the chip made of a conventional Si substrate is ≈5 times higher.

### TIRF results

**Figure 5:**
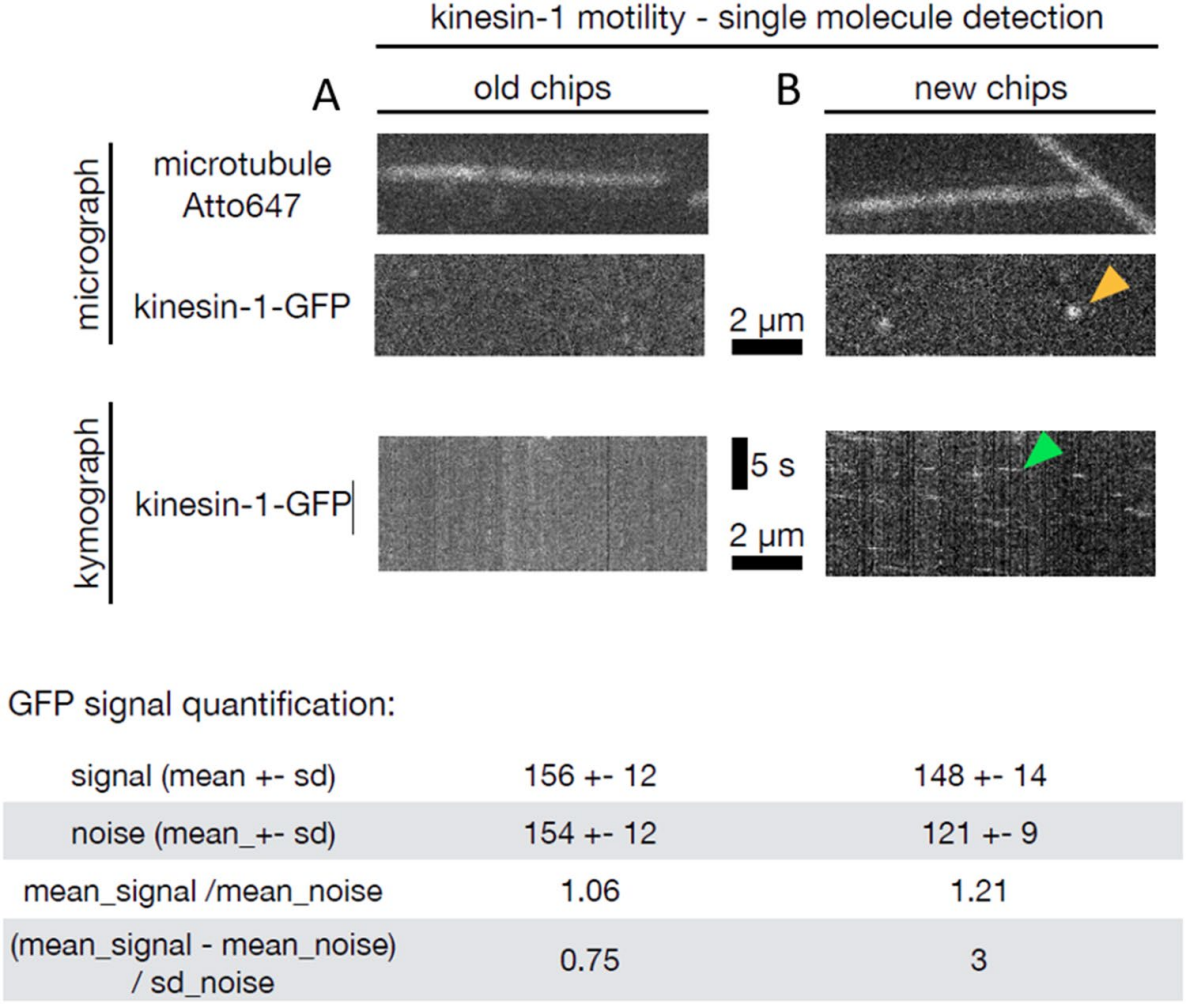
TIRF results from the (left) chip made from the conventional substrate and (right) an improvement once the SOI-based chip was used. Microtubule is detectable in both chips. Single kinesin-1-GFP molecules (in micrograph and kymograph indicated by orange and green arrowheads, respectively) can be detected only in the SOI-based chips.

We tested single-molecule detection in these chips using an established assay^38^, where microtubules are specifically attached to a surface and the motility of single fluorescently labeled kinesin-1 molecular motors along these microtubules is visualized using TIRF microscopy. A single kinesin-1 motor is a homodimer yielding a relatively low fluorescent signal originating from two attached molecules of green fluorescent protein (GFP). After attaching the microtubules to the chips, we added kinesin-1-GFP at = 2.5 nM concentration, imaged both the microtubules and the kinesin-1, and compared conventional substrate and SOI-based chip compatibility with the assay. Microtubules could be visualized in both chip versions using excitation with light of 640 nm wavelength (Fig. 5). However, the motility of a single GFP-labeled kinesin-1 requiring illumination with 488 nm wavelength could only be successfully visualized using SOI-based chips (Fig. 5 right). This is due to the low level of the signal of single kinesin-1 molecules and the elevated background noise level in the conventional chips (Table 1). The results confirm the suppression of background noise in SOI-based chips and show the capability to use the SOI-based chips for single-molecule detection assays using TIRF microscopy.

**Table 1:** Signal and noise parameters from both chip types derived from single-molecule motility TIRF assay.

	Conventional chip	SOI-based chip
GFP signal (mean ± σ)	156 ± 12	148 ± 14
Background noise (mean ± σ)	154 ± 12	121 ± 9
Mean signal/mean noise	1.01	1.22
(Mean signal − mean noise)/σ noise	**0.17**	**3**

Significant values are in bold.

The most significant parameter improvement is an increase of a (mean signal − mean noise)/σ noise ratio by = 17.6 × allowing the determination of motility of a single GFP-labeled kinesin-1 requiring illumination of 488 nm wavelength.

## Conclusion

In this contribution, we analyzed the properties of high-end optical filters concerning single-molecule imaging using microfluidics chips.

We would like to stress that the last fabrication step before the anodic bonding closing the microfluidic system was the Si thermal oxidation to grow a SiO_2_ layer with thickness of 5 nm conducted at 900 °C in dry O_2_ ambient to define the chamber surface properties. This step in that environment removes all traces of chemicals possibly bonded to the original Si surface. Si as well as the glass used for anodic bonding both do not exhibit any autofluorescence and the only difference between the two different microfluidic chip (Si and SOI-based) is the bottom topography. The rest is identical. Thus whatever the effect is, the bottom line is that it is related to the surface topography and removal of this topography significantly improves the fluorescent measurement (imaging) properties.

We have found that high-resolution imaging, such as was used for single-molecule motility determination using the TIRF technique, does not achieve expected result while using microfluidics chips made by the conventional technique using a bulk silicon substrate, due to possible various effects related to the rough bottom of the structure. We studied this effect and demonstrated the solution that replacing the conventional Si substrate with an SOI substrate guarantees a flat bottom of the microfluidic chip, allowing the TIRF technique to maximize its potential by determining single-molecule motility using illumination at a 488 nm wavelength. Fairly expensive SOI substrates can probably be replaced with conventional Si wafers and allow microfluidic chamber etching through a suitable smoothening technique such as the one based on HF/HNO_3_ etching^39^. Regardless, the cost of the original substrate is typically rather marginal in comparison with chip fabrication and especially with its subsequent utilization including single-molecule testing, which justifies this high substrate cost.

## Data Availability

There is no data availability section present on system and data can be obtain based on a reasonable request.
